# *AW*BAT^TM^: Early Clinical Experience

**Published:** 2010-03-15

**Authors:** Victoria B. Vandenberg

**Affiliations:** Vandenberg Associates, Dana Point, Calif

## Abstract

**Objective:** The purpose of this article is to describe the early clinical experience with *AW*BAT. **Methods:** Burn patients requiring (1) donor sites or (2) treatment of a superficial burn wound injury were treated. A total of 45 patients with 69 distinct wounds were included. *AW*BAT^TM^-D was evaluated in donor sites and *AW*BAT^TM^-S was evaluated in superficial partial-thickness burns. Days to healing, pain, hematoma/seroma formation, and infection were noted. Ease of application, adherence, transparency, and physical adaptability details were collected. **Results:** Average period to healing of donor sites treated with *AW*BAT-D (*n*=22 patients with *n*=26 wounds) was 11.2 days, σ =1.95, with a range of 8–15 days and a median of 11 days. Pain rating at 24 hours was 1.2, σ =0.43 (*n*=18) and at 48 hours mean was 1.2, σ =0.46 (*n*=15). Average period to healing of superficial burns treated with *AW*BAT-S (*n*=15 patients with *n*=18 wounds) was 8.1 days, σ =2.48, with a range of 5–13 days and a median of 7 days. Pain rating at 24 hours was 1.5, σ =0.85 (*n*=10) and at 48 hours mean was 1.75, σ =0.89 (*n*=8). There was zero incidence of hematoma/seroma. No infections were seen. Results indicate that *AW*BAT was easily applied with good initial adherence. It was noted to be transparent, conformant, and pliable. **Discussion:** Early experience demonstrates that *AW*BAT performs well on donor sites and superficial partial-thickness burns and delivers the desired attributes of a temporary skin substitute including good adherence, infection control, transparency, adapatability, and pain control.

*AW*BAT^TM^ is a newly designed advanced wound care product intended to satisfy the desired attributes of a temporary skin substitute. *AW*BAT consists of a thin silicone-nylon membrane with porcine type 1 collagen peptides embedded in the nylon. *AW*BAT is manufactured in such a way that the continuity of the 3-dimensional nylon structure over the entire surface of the dressing is unharmed. *AW*BAT was cleared by the Food and Drug Administration in February 2009 and is available in 3 configurations: *AW*BAT-D, intended for donor sites after hemostasis has been established; *AW*BAT-S, intended for clean, superficial burn wounds; and *AW*BAT-M for use as a protective covering for meshed autografts. *AW*BAT was evaluated in an early clinical experience program between May and July 2009. Thirteen surgeons at 8 burn centers across the United States participated in this program.

*AW*BAT-D was evaluated for use on donor sites, and *AW*BAT-S was evaluated on clean, superficial burns.

Two configurations of *AW*BAT^TM^ are reported in this article. Both *AW*BAT-D and *AW*BAT-S consist of a thin silicone-nylone membrane with porcine type 1 collagen peptides embedded in the nylon. The primary difference in the 2 configurations is the porosity. *AW*BAT-D is approximately 9% porous, whereas *AW*BAT-S is approximately 5.5% porous.

## METHODS

*AW*BAT-D was evaluated in patients requiring the harvest of donor sites for skin grafting of full-thickness burns, and *AW*BAT-S was evaluated in patients requiring treatment of clean, superficial burns. Each group will be described in detail.

## Donor sites

Following surgical preparation and draping, donor sites were harvested using a dermatome. Depth of grafts ranged from 0.008 in to 0.015 in. Both tumescent and nontumescent techniques were used. Once harvested, hemostasis was achieved using a variety of techniques including epinephrine soaks and fibrin sealants. *AW*BAT-D was then placed on the hemostatic wound with the nylon/collagen (dull) side down and silicone (shiny) side up. Anchoring one edge, the product was smoothed over the wound to minimize wrinkling. Various anchoring methods including steri-strips, surgical staples, sutures, and liquid adhesives were used. An outer absorptive dressing was then placed over the secured *AW*BAT-D. Various products were used including an Exu-Dry^TM^ pad or an ABD pad. The outer dressings were held in place with a Kerlix^TM^ roll followed by an ace wrap. Patients were examined at 24 to 48 hours and evaluated for hematoma/seroma formation, level of pain, and signs of infection. In addition to these clinical parameters, the product was evaluated for physical characteristics including ease of application, adherence, and transparency. Patients were then followed until the donor sites were healed to document the number of days it took to heal the wound.

## Superficial burn wounds

Superficial partial-thickness burns were treated with *AW*BAT-S. First, blisters, loose skin, and debris were removed from the burn wound. *AW*BAT-S was placed onto the wound with the nylon/collagen (dull) side down and silicone (shiny) side up. It was gently stretched and smoothed over the open wound to minimize wrinkling. Various anchoring methods were used including steri-strips, surgical staples, sutures, and liquid adhesives. An outer absorptive dressing was then placed over the secured *AW*BAT-S. Various products were used including an Exu-Dry^TM^ pad or an ABD pad. The outer dressings were held in place with a Kerlix^TM^ roll followed by an ace wrap. Patients were examined at 24 to 48 hours and evaluated for hematoma/seroma formation, level of pain, and signs of infection. In addition to these clinical parameters, the product was evaluated for physical characteristics including ease of application, adherence, and transparency. Patients were then followed until the donor sites were healed in order to document the number of days it took to heal the wound.

## Evaluation

At the time of application, the product was evaluated for a number of physical characteristics and qualities including transparency, adherence, and ease of application. In the following days, the wounds were evaluated for hematoma or seroma formation, as well as signs of infection. The primary end point was days to healing of the wound. Inpatients were evaluated for healing daily. Outpatients were evaluated at routine clinic visits. All patients were asked to evaluate their pain on a scale of 1 to 10, with 1 defined as no pain and 10 defined as being the worst pain ever experienced.

## RESULTS

The initial clinical experience in donor sites and superficial partial-thickness burns consisted of 45 patients with a total of 69 wounds. *AW*BAT-D was used in 26 patients on a total of 34 wounds. *AW*BAT-S was used in 19 patients on a total of 35 wounds.

## Donor sites

The experience using *AW*BAT-D for donor site wounds resulted in a total of 22 evaluable patients with 26 wounds. Data from 4 patients are not included as 2 patients had the *AW*BAT-D removed at day 6 per burn center donor site protocol, 1 patient expired, and 1 patient was lost to follow-up after surgery. Results are summarized in Table 1.

**Figure 1:**
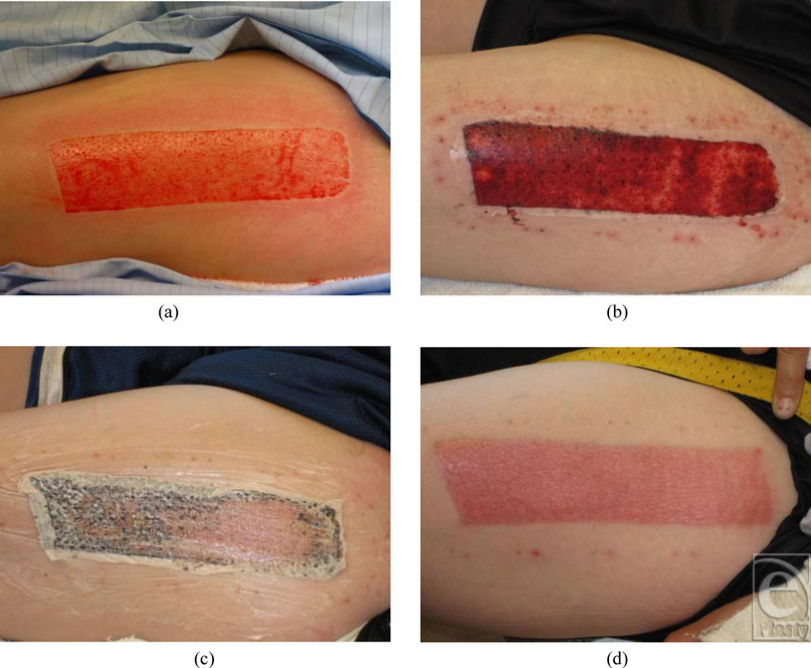
(a) Harvested donor site, (b) postoperative day 3, *AW*BAT-D well adhered. Staples have been removed. (c) Petrolatum-based product was applied to loosen *AW*BAT-D on day 10, and (d) day 13 result. Used with permission from Aubrey, Inc.

The range of days to healing was 8 to 15, the mean days to healing was 11.3 (SD = 1.95), and median was 11.0 days. Pain was evaluated at 24 and 48 hours. At 24 hours, the mean pain rating was 1.2 (SD = 0.43) (*n* = 18), and at 48 hours, the mean was 1.2 (SD = 0.46) (*n* = 15). No hematomas or seromas were noted. Importantly, there were no infections reported. The physical properties of *AW*BAT-D were also evaluated. Characteristics evaluated included the ease of application and the initial adherence of the product. In the 34 wounds covered with *AW*BAT-D, the ease of application was excellent (*n* = 27) and good (*n* = 7) in 100% of the patients treated. The initial adherence was excellent (*n* = 17) and good (*n* = 17) in 100% of the patients treated. In addition, *AW*BAT-D was consistently noted to be transparent, conformant to the wound, and pliable (see Fig 1).

## Superficial partial-thickness burn wounds

The experience with *AW*BAT-S resulted in a total of 15 evaluable patients with 21 wounds. Data from 4 patients is not included as 1 patient with excellent initial results of good adherence of the *AW*BAT-S over a 24% TBSA removed all the product at day 2 while in delirium tremens (DTs), 1 patient expired, 1 patient was lost to follow-up after surgery, and 1 patient had *AW*BAT-S removed by the surgeon due to nonadherence on a deep wound. Results are summarized in Table 2.

The range of days to healing was 5 to 13, the mean days to healing was 8.1 (SD = 2.48), and the median was 7.0 days. Pain was evaluated at 24 and 48 hours using a pain scale of 1 to 10. At 24 hours, the mean pain rating was 1.5 (SD = 0.85) (*n* = 10) and at 48 hours, the mean was 1.75 (SD = 0.89) (*n* = 8). No hematomas or seromas were noted. Importantly, there were no infections reported.

**Figure 2:**
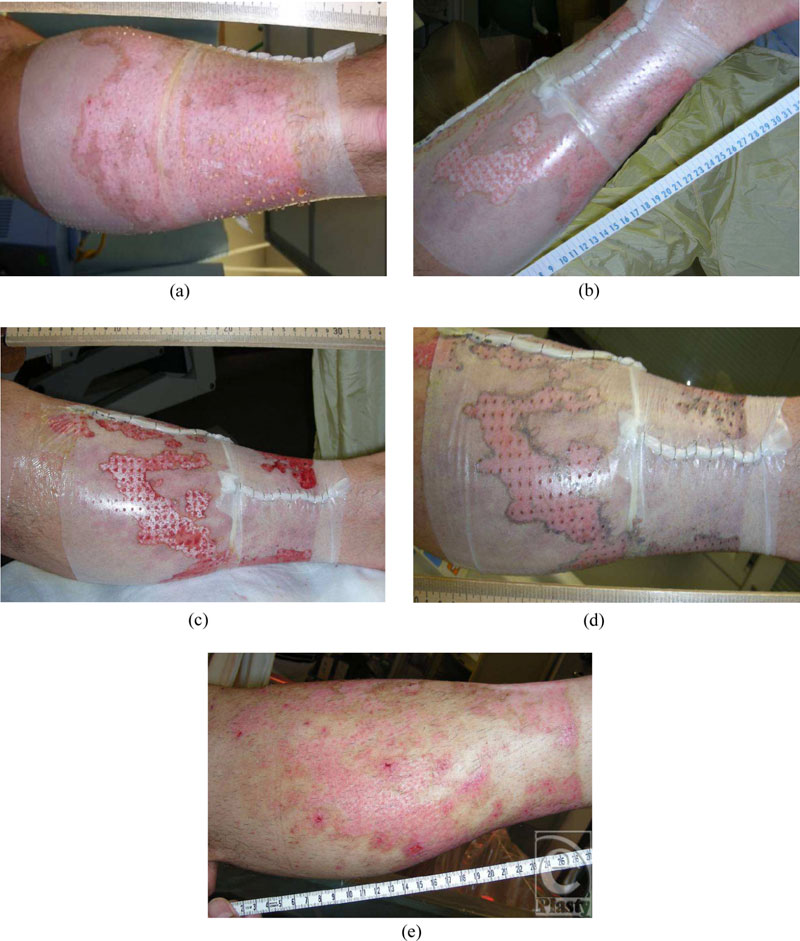
(a) Immediately following application of *AW*BAT-S to a superficial partial-thickness burn. Note fluid passing through pores. (b) Product is well adhered at day 1, (C) day 3, no fluid accumulation under dressing, (D) showing signs of healing at day 8, (e) day 13, patient returned to clinic, healed. Used with permission from Aubrey, Inc.

The physical characteristics evaluated included the ease of application and the initial adherence of the product. Of the 35 wounds covered with *AW*BAT-S, the ease of application was excellent (*n* = 15) or good (*n* = 15) on 86% of the patients treated and fair (*n* = 5) in 13% of patients treated. The initial adherence was excellent (*n* = 9) or good (*n* = 26) in 100% of patients. In addition, *AW*BAT-S was consistently noted to be transparent, conformant to the wound, and pliable (see Fig 2).

Further examination of the data was done to see what impact anesthesia and tumescence had on healing rates. Patients undergoing surgery for donor sites all had general anesthesia. Approximately half of the donor sites were harvested using a tumescent technique. The average time to healing for the tumesced group was 11.3 days whereas the nontumesced group's average time to healing was 11.0 day. Patients with superficial burns received either general anesthesia or Ketamine. The average time to healing for the general anesthesia group was 7.5 days and for the Ketamine group it was 9.3 days.

## DISCUSSION

Burn injury results in the loss of integrity of the skin. The need for an ideal skin substitute is well understood while the quest for an ideal skin substitute has been ongoing for decades. Since 1979, when the Food and Drug Administration cleared the first biologically based wound dressing (Biobrane) the search has continued. The ideal properties of a synthetic wound dressing or biologic skin substitute have been outlined by numerous authors.^1^^-^4 The desirable properties include rapid and sustained adherence, moisture permeability, no increase in infection, conformance to surface irregularities, transparency, physical adaptability, minimization of patient discomfort, safety, and stability.

Early clinical experience data with *AW*BAT were collected from 8 burn centers across the United States. The product evaluation process addressed the desired qualities of an advanced wound care product for use in burn centers.

*AW*BAT was noted to have excellent to good initial adherence in 92.8% of applications. Complications such as hematoma/seroma formation under biological dressings can complicate the postoperative course, leading to the need for evacuation of the fluid or removal of the dressing. Fluid collection can inhibit adherence and may also lead to infection. *AW*BAT is highly porous and features nylon that provides a contiguous 3-dimensional structure. It allows fluid to pass through the pores thereby minimizing fluid accumulation once placed on the wound. Fluid passes through the *AW*BAT into an outer absorptive dressing as noted in Figure 2A.

The physical characteristics of *AW*BAT were also evaluated. This included transparency, conformance of the product to the wound, and pliability. *AW*BAT was consistently noted to be transparent, conformable to the wound, and pliable.

Among the numerous clinical observations made during the program, perhaps the most clinically significant was the rapid healing of donor site wounds. Healing was observed in 8 to 15 days. On some wounds, particularly outpatients, it was observed that the material could have been removed sooner because of this rapid healing process (Fig 1C). When removing *AW*BAT-D from a wound that appeared to be healed, application of a petrolatum-based product or soaking was effective. In addition, because of the rapid healing, it should be noted that the range of days to healing might be overstated in the program statistics.

Another important clinical observation was that without exception *AW*BAT was used as a primary, single application dressing. In all cases, it remained on the wound until healing was complete. Single application dressings typically reduce the need for multiple dressing changes and thereby the associated costs and patient discomfort. Cost data were not specifically tracked in this program; therefore, no specific conclusions can be drawn regarding cost savings.

Data were collected on the patient's perception of pain from those patients who were able to verbalize. The data clearly indicate that patients treated with *AW*BAT had very low postoperative pain scores. In addition, there were many anecdotal comments from the burn care teams regarding the minimal level of pain in patients treated with *AW*BAT.

Evaluation of *AW*BAT is continuing in multicenter, comparative trials.

## SUMMARY

*AW*BAT performs well as a donor site dressing and as a treatment of superficial partial-thickness burns. Early evaluation indicates that *AW*BAT delivers the desired attributes of an advanced wound burn dressing, including good adherence, moisture control, transparency, and physical adaptability. Importantly, *AW*BAT performed strongly in the areas of infection control and pain management.

## Figures and Tables

**Table 1:** Summary of performance of *AW*BAT-D on donor sites

			Days to healing				
*AW*BAT-D	Total, *N*	Evaluable, *n*	Range	Mean	Median	Infection	Hematoma/seroma
Patients	26	22					
Wounds	34	26	8–15	11.3 ± 1.95	11.0	None	None

**Table 2:** Summary of *AW*BAT-S performance on superficial partial-thickness burns

			Days to healing				
*AW*BAT-S	Total, *N*	Evaluable, *n*	Range	Mean	Median	Infection	Hematoma/seroma
Patients	19	15					
Wounds	35	21	5–13	8.1 ± 2.48	7.0	None	None
